# Comparison of modified U-shaped and inverted L-shaped medial capsulorrhaphy in hallux valgus surgery: a prospective, randomized controlled trial of 75 patients

**DOI:** 10.1186/s13018-023-03799-1

**Published:** 2023-04-24

**Authors:** Xiaohua Wei, Xiong Liu, Peng Zhang, Shifeng Liu

**Affiliations:** 1 Department of Traumatic Orthopaedic, Dongguan Eighth People’s Hospital, Dongguan, Guangdong China; 2 Orthopaedic Center, The First Dongguan Affiliated Hospital, Guangdong Medical University, Dongguan, Guangdong China

**Keywords:** Medial capsulorrhaphy, Distal soft tissue procedure, Hallux valgus

## Abstract

**Purpose:**

The purpose of this study was to report a modified U-shaped medial capsulorrhaphy and compare its clinical and radiological differences with an inverted L-shaped capsulorrhaphy in hallux valgus (HV) surgery.

**Methods:**

A prospective study of 78 patients was performed between January 2018 and October 2021. All patients underwent chevron osteotomy and soft tissue procedures for HV, and the patients were randomly separated into 2 groups according to the medial capsule closing techniques: a modified U-shaped capsulorrhaphy (group U) and an L-shaped capsulorrhaphy (group L). All patients were followed for at least a year. The preoperative and follow-up data were collected for each patient and included patient demographics, weight-bearing radiographs of the foot, the active range of motion (ROM) of the first metatarsophalangeal (MTP) joint and the American Orthopedic Foot and Ankle Society (AOFAS) forefoot score. Mann–Whitney *U* test was used for the comparison of the postoperative measures between the groups.

**Results:**

In total, 75 patients with 80 affected feet met the inclusion criteria, with 38 patients (41 feet) in group U and 37 patients (39 feet) in group L. One year after surgery, the mean hallux valgus angle (HVA), intermetatarsal angle (IMA), and AOFAS score in group U improved from 29.5 to 7.1, from 13.4 to 7.1, and from 53.4 to 85.5, respectively. The mean HVA, IMA, and AOFAS score in group L improved from 31.2 to 9.6, from 13.5 to 7.9, and from 52.3 to 86.6, respectively. Comparing the 1-year postoperative measures between the 2 groups, a significant difference was found in HVA (*P* = 0.02), but not found in IMA and AOFAS score (*P* = 0.25 and *P* = 0.24, respectively). The mean ROM of the first MTP joint was 66.3 degrees preoperatively and 53.3 degrees at the 1-year follow-up in group U, while 63.3 and 47.5 in group L. The degrees of ROM after 1 year in group U were better than those in group L (*P* = 0*.*04).

**Conclusion:**

Compared to the inverted L-shaped capsulorrhaphy, the modified U-shaped capsulorrhaphy provided a better ROM of the first MTP joint; at 1 year following surgery, the modified U-shaped capsulorrhaphy maintained the normal HVA better.

## Introduction

To correct hallux valgus (HV) deformities, bony osteotomies are commonly used. However, soft tissue structures also play an important role in the etiology, progression, and treatment of HV [1]. Bonny osteotomy typically corrects the intermetatarsal angle (IMA), whereas the hallux valgus angle (HVA) can be maintained by distal soft tissue procedures [2]. A suitable combination of an osteotomy and a soft tissue procedure is usually required to achieve balance of the joint [3]. The aim of the distal soft tissue operation is to restore the first metatarsophalangeal (MTP) joint’s physiologic balance of capsular, ligamentous, and muscular structures. Several studies have found that lateral soft tissue release is an effective surgical procedure for increasing correction in HV surgery [3–5]. However, to the best of our knowledge, minimal attention has been given to the medial soft tissue procedure. Given the relative lack of reporting specifically related to this, the purpose of this study was to report a modified U-shaped medial capsulorrhaphy and compare its clinical and radiological differences with an inverted L-shaped capsulorrhaphy.

## Patients and methods

### Materials and methods

A prospective, randomized controlled trial of 78 patients who underwent operative treatment for mild to moderate HV from January 2018 to October 2021 was performed. The indications for surgery were that a symptomatic patient had conservative treatment that was ineffective. The inclusion criteria were as follows: ① age ranging from 18 to 70 years old and ② mild to moderate HV deformity (HVA of 20 to 40 degrees and IMA of 11 to 16 degrees [6]). The exclusion criteria were as follows: ① hallux rigidus and ② rheumatoid arthritis or osteoarthritis on the first MTP joint. This study was approved and conducted in accordance with the protocol of the Institutional Medical and Ethics Committee of Dongguan Eighth People’s Hospital.

All patients were treated with lateral soft tissue release, chevron osteotomy, and medial capsulorrhaphy for HV, and the patients were separated into 2 groups according to the randomization approach. The randomization was achieved by using sealed envelopes. Each of the envelopes was prepared and contained one of the surgical techniques, and all 78 envelopes were stored in a box. These envelopes were drawn in the operating room before the surgery. All sealed envelopes were prepared by an independent person. A modified U-shaped capsulorrhaphy was implemented for the patients in group U, and an L-shaped capsulorrhaphy was performed for the patients in group L. All surgical procedures were performed by a single surgeon.

The preoperative and follow-up data were collected for each patient, which included patient demographics (age, sex, general physical condition). Degrees of range of motion (ROM) of the first MTP joint, routine weight-bearing anteroposterior (AP), and lateral radiographs (including the HVA and IMA) were obtained preoperatively, as well as at 12 months after surgery. The HVA and IMA were measured by computer-assisted measurement. The ROM of the first MTP joint was obtained by using a goniometer and was taken while moving the first MTP joint from maximum plantar flexion to maximum dorsiflexion. Clinical results were assessed using the American Orthopedic Foot and Ankle Society (AOFAS) forefoot score before surgery and 12 months postoperatively.

### Surgical technique

The patient was given spinal or general anesthesia based on the anesthesiologist’s recommendation. The patient was placed in a supine position, and a lower extremity tourniquet was applied for better surgical vision. During the operation, a lateral soft tissue release was performed in the same manner as described by Schneider [7]. After the lateral soft tissue release was completed, the medial capsule was opened, the medial pseudoexostosis was excised, and a 60-degree chevron osteotomy was performed with an oscillating saw based on the distal metatarsus. The distal fragment was laterally translated until the IMA was reduced. The osteotomy was fixed with a 3.5-mm-diameter fully threaded cancellous screw, and the extra medial eminence was removed.

For the L-shaped medial capsulorrhaphy technique, before chevron osteotomy, a vertical capsular incision (short arm) was first performed proximal to the base of the proximal phalanx. Then, a dorsomedial incision extending from the interphalangeal joint to the midshaft of the first metatarsal (long arm) was made in the capsule parallel to the first metatarsal, creating a plantarly based capsular flap. Third, after the extra medial eminence was removed from the metatarsal shaft during the chevron osteotomy, the redundant edges of the capsular flap were resected carefully, and the tip was sutured to the capsule piece distal to the first proximal phalanges [8].

**Fig. 1:**
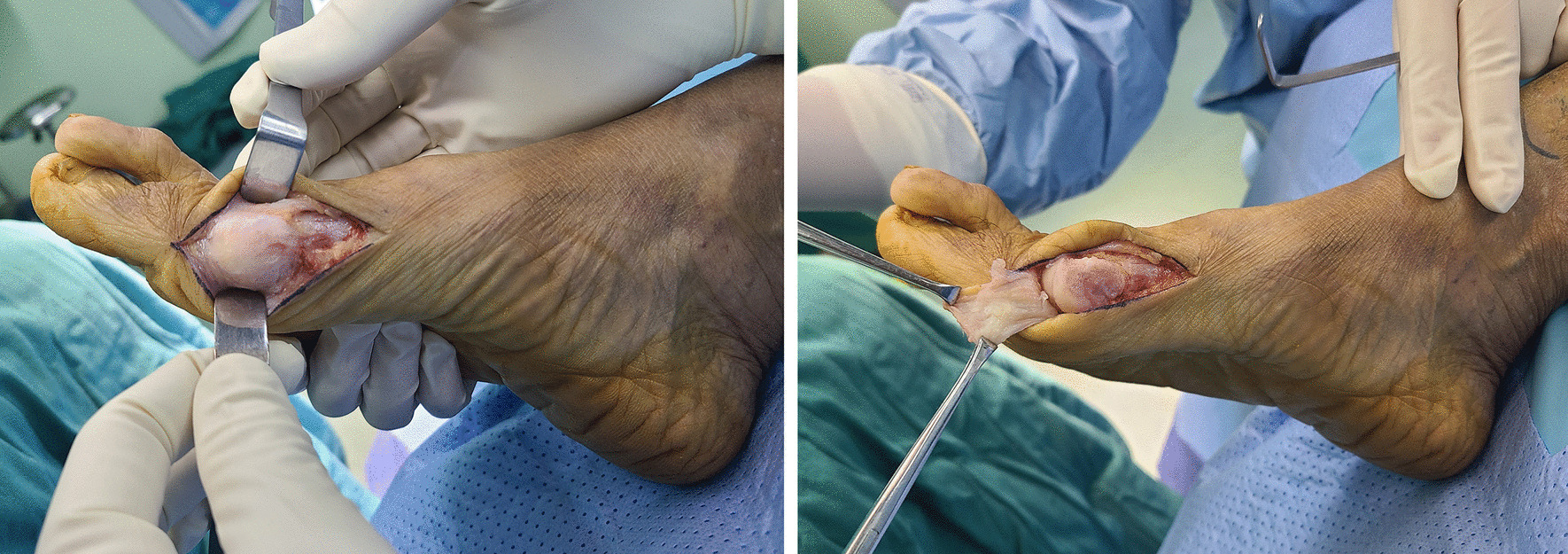
The U-shaped capsular flap preparing. The medial capsule was exposed (left) and a transversal U-shaped capsular flap is formed (right) during the U-shaped medial capsulorrhaphy procedure

**Fig. 2:**
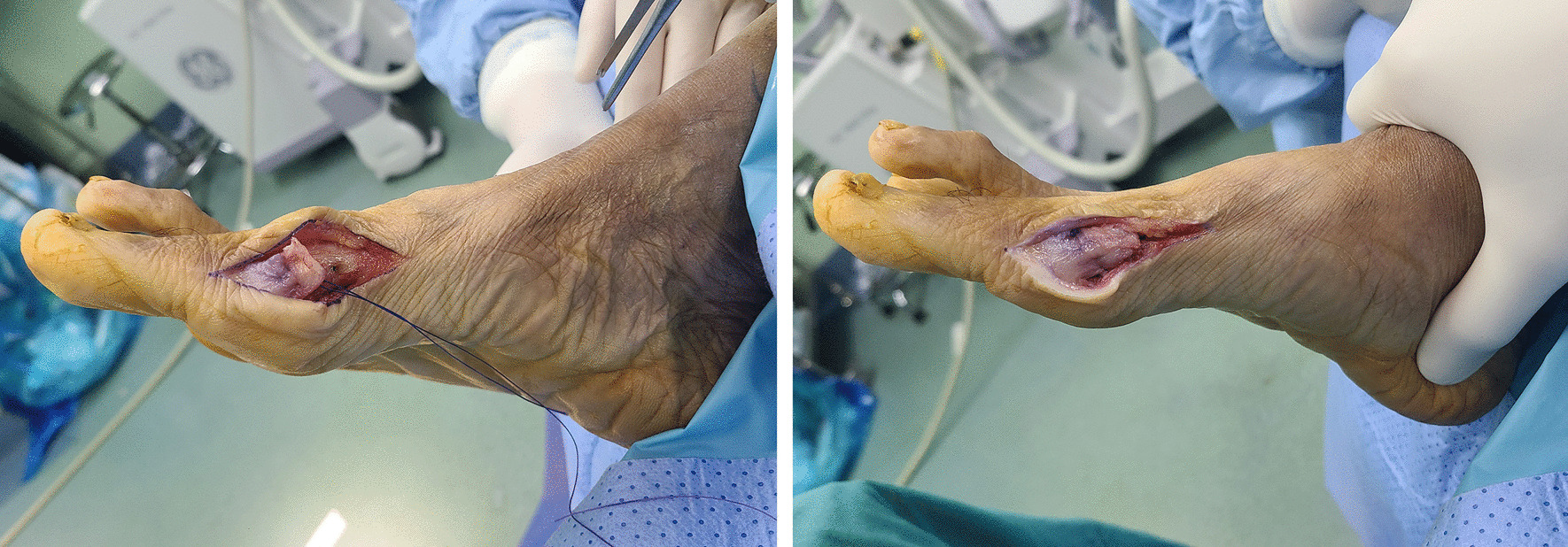
The U-shaped capsular flap fixation. A 1.2-mm hole was drilled perpendicular from dorsal to plantar of the distal fragment. A resorbable-coated VICRYL thread was used to stitch the medial capsule and collateral ligament in a running-locking interrupted fashion. One end of the proximal suture was pulled through the hole and knot it with the other end until the great toe became straight (left). Resorbable thread was used to reinforce the terminal of medial soft tissue (right)

In the modified U-shaped group, the medial capsule and collateral ligament were dissected completely from the distal metatarsal, and then a transverse U-shaped capsular flap was formed (Fig. 1). The medial eminence was removed flush from the first metatarsal shaft. After the lateral soft-tissue release and chevron osteotomy were completed, a transosseous trimming suture was used for medial capsule and collateral ligament repositioning. A 1.2-mm hole was drilled perpendicular from dorsal to plantar of the distal fragment (approximately 0.5 cm near the osteotomy line). A 1# resorbable-coated VICRYL (Ethicon, VCP359) suture was used to stitch the medial capsule and collateral ligament in a running-locking interrupted fashion. One end of the proximal suture was pulled through the hole, and it was knotted with the other end until the great toe became straight (Fig. 2). Excessive tightening of the medial structures should be avoided to prevent overcorrection or varus malalignment. Then, both incisions were closed.

### Postoperative care

A gauze dressing was applied at the first web space for 2 weeks postoperatively. After the sutures were removed, a 12-mm-thick spacer was used for another 4 weeks. Care was taken to avoid pronating the toe or forcing it into varus. The patients were encouraged to ambulate with a heel-weight-bearing shoe, and early active and passive mobilization exercises for the toe were advised after surgery. The wearing of shoes without restrictions was recommended at 2 months postsurgery.

### Data analysis

The radiographic reviews and measurements were obtained independently by two experienced orthopedic surgeons, and all statistical analyses were performed using SPSS (v.23.0, SPSS Inc, Chicago, IL, USA). Descriptive statistics were defined as means and standard deviations (± SD). Mann–Whitney *U* test was used for the comparison of continuous variables, and categorical variables were analyzed with the Chi-square test or Fisher’s exact test. *P* < 0.05 was considered significant.

## Results

Three patients were excluded due to inadequate follow-up, with adequate follow-up defined as at least 12 months. In total, 75 patients with 80 affected feet met the inclusion criteria, and there were 38 patients (41 feet) in group U and 37 patients (39 feet) in group L. The average age was 43.6 ± 15.8 for group U and 45.3 ± 16.3 for group L. The ratio of male patients was 21.1% (8) and 24.3% (9), respectively. A comparison of weight showed no statistically significant difference between the two groups. The distribution of sexes, ages, and sides of affected limb demonstrated no significant difference (Table 1).

**Table 1:** Baseline characteristics of the patients in two groups

Variable	Group U (*n* = 38)	Group L (*n* = 37)	*P* values
Age (years) Mean ± SD	43.6 ± 15.8	45.3 ± 16.3	0.65
Gender (Male/female)	8/30	9/28	0.74
Side (Left/right)	24/17	18/21	0.27
Weight (kg) Mean ± SD	57.8 ± 5.6	59.0 ± 4.7	0.35

SD, standard deviation

The postoperative HVA and IMA improved significantly in both groups. In group U, the mean HVA was 29.5 (range, 20 to 40) degrees preoperatively and 7.1 (range, 2 to 16) degrees at the 1-year follow-up. The mean IMA was 13.4 (range, 11 to 16) degrees preoperatively and 7.1 (range, 3 to 12) degrees at the 1-year follow-up. In group L, the mean HVA was 31.2 (range, 20 to 40) degrees preoperatively and 9.6 (range, 3 to 20) degrees at the 1-year follow-up. The mean IMA was 13.5 (range, 11 to 16) degrees preoperatively and 7.9 (range, 4 to 13) degrees at the 1-year follow-up. Comparing the degrees of HVA and IMA at 1 year after surgery, the statistical value of IMA was insignificant (*P* = 0*.*25), but a statistically significant difference in HVA was observed between the 2 groups (*P* = 0*.*02, Table 2).

**Table 2:** Radiological data of both groups

	Group U	Group L	*P* value
*HVA*			
Preoperative	29.5 ± 5.3	31.2 ± 6.1	0.17
12 months	7.1 ± 4.5	9.6 ± 4.8	0.02
*IMA*			
Preoperative	13.4 ± 1.8	13.5 ± 1.5	0.76
12 months	7.1 ± 3.1	7.9 ± 2.4	0.25

HVA, hallux valgus angle; IMA, intermetatarsal angle

The mean ROM of the first MTP joint was 66.3 degrees preoperatively and 53.3 degrees at the 1-year follow-up in group U, while the mean ROM of the first MTP joint was 63.3 degrees preoperatively and 47.5 degrees at the 1-year follow-up in group L. The degrees of ROM after 1 year in group U were better than those in group L (*P* = 0*.*04). The AOFAS forefoot scores were 53.4 preoperatively and 85.5 at the 1-year follow-up in group U and 52.3 and 86.6, respectively, in group L. Significant improvement was achieved for both groups, but neither group showed any statistically significant differences (*P* = 0*.*24, Table 3).

**Table 3:** ROM of the first MTP joint

	Group U	Group L	*P* value
*ROM*			
Preoperative	66.3 ± 11.8	63.3 ± 12.2	0.24
12 months	53.3 ± 11.9	47.5 ± 11.8	0.04
*AO-FAS score*			
Preoperative	53.4 ± 7.7	52.3 ± 8.0	0.78
12 months	85.5 ± 3.9	86.6 ± 3.9	0.24

AOFAS, American Orthopedic Foot and Ankle Society; MTP, metatarsophalangeal; ROM, range of motion

There was also a very low incidence of 1-year postoperative complications, including recurrent asymptomatic HV in 4 of the patients from group L and 1 of the patients from group U (defined as HVA higher than 15 degrees [9]). In group L, there were 2 patients with first MTP joint stiffness (defined as ROM less than 30 degrees [10]) and one patient with hallux varus (defined as HVA less than -3 degrees) in group U.

## Discussion

According to our study, the HVA and IMA in both groups returned to normal angles postoperatively. After 12 months of follow-up, the HVA had significantly changed in group L compared with group U, and recurrence of the HV deformity occurred in 4 patients in group L and 1 patient in group U. Compared with the inverted L-shaped capsulorrhaphy, the modified U-shaped capsulorrhaphy is less likely to result in a loss of HVA and help prevent the recurrence of HVA. Normal activity of the first MTP joint is one of the main objectives of HV surgery. In our study, the mean ROM of the first MTP joint was 53.3 ± 11.9 degrees at the 1-year follow-up in group U and 47.5 ± 11.8 degrees in group L. The degrees of ROM after 1 year in group U were better than those in group L. This result suggests that compared with inverted L-shaped capsulorrhaphy, modified U-shaped capsulorrhaphy is less likely to result in ROM loss in the first MTP joint.

In our study, compared with inverted L-shaped capsulorrhaphy, modified U-shaped capsulorrhaphy could maintain the HVA better after 1 year of follow-up. The result could be explained by the fact that after the lateral soft tissue is released, the modified U-shaped capsulorrhaphy technique can shorten the extended medial collateral ligament (MCL), tighten the capsule, and then maintain the balance of the first MTP joint. Another study comparing clinical and radiographic differences between longitudinal capsulorrhaphy and inverted L-type capsulorrhaphy in patients diagnosed with HV showed that inverted L-type capsulorrhaphy was more effective in correcting the HVA [11].

Schneider [7] suggested that development of an incongruous joint leads to elongation of the MCL and capsular structures, and the medial reefing procedure can shorten the MCL and decrease the HVA. We also obtained a similar result as described in that study. The other result of our study is that the first MTP joint movement restriction from modified U-shaped capsulorrhaphy is significantly less than that from inverted L-shaped capsulorrhaphy. The likely causes are as follows: ① The suture and the knots are far away from the articular surface. ② The tightening force of the tensioning sutures at the joint is parallel to the MCL, which can improve the joint’s physiological and anatomical construction. A previous cadaver study comparing the immediate effect of the Y-shaped and inverted L capsulorrhaphy methods on the ROM of the first MTP joint revealed that Y-shaped capsulorrhaphy produces significantly less joint stiffness than inverted L capsulorrhaphy [8].

There was 1 case of recurrence in group U and 4 of the patients from group L at the 1-year follow-up. HV recurrence can result from insufficient lateral release, inappropriate sesamoid realignment, or poor medial capsule repair, so lateral and medial soft tissue imbalance is the common reason of recurrence [12]. One patient experienced hallux varus in group U. The complication of hallux varus may be caused by the excessive fixation of the medial soft tissue, so care should be taken to avoid gross fixation of the medial capsular flap.

There are also some limitations to this study. First, when evaluating the ROM of the first MTP joint, which was taken while moving the first MTP joint from maximum plantar flexion to maximum dorsiflexion, we did not distinguish dorsiflexion and plantar flexion movement of the first MTP joint. Second, the sample size was small, and the follow-up time was short term. A larger cohort and a longer follow-up are required to evaluate the results verifiably.

## Conclusion

Compared to the inverted L-shaped capsulorrhaphy, modified U-shaped capsulorrhaphy provided a better ROM of the first MTP joint and maintained the normal HVA better 1 year following surgery; it could be a better choice for medial soft tissue reconstruction in HV surgery.

## Data Availability

The datasets used or analyzed during the current study are available from the corresponding author on reasonable request.
